# Hydrogen Permeation Resistance of PVDF–Graphene Nanocomposite Coatings for Metallic Pipelines

**DOI:** 10.3390/polym17162262

**Published:** 2025-08-21

**Authors:** Mohammed M. Aman, Bashar S. Mohammed, Ahmad Mahamad Al-Yacouby

**Affiliations:** Department of Civil and Environmental Engineering, Universiti Teknologi Petronas, Seri Iskandar 32610, Malaysia; mohammed_22012355@utp.edu.my (M.M.A.); ahmad.alyacouby@utp.edu.my (A.M.A.-Y.)

**Keywords:** hydrogen, embrittlement, inhibition, coating, graphene, nanocomposite

## Abstract

Hydrogen-induced steel embrittlement imposes a technical difficulty in facilitating effective and safe hydrogen transportation via pipelines. This investigative study assesses the potency of polyvinylidene fluoride (PVDF)–graphene-based composite coatings in the inhibition of hydrogen permeation. Spin coating was the method selected for this study, and varying graphene concentrations ranging from 0.1 to 1wt% were selected and applied to 306 stainless steel substrates. A membrane permeation cell was used in the evaluation of hydrogen permeability, while the impact of graphene loading on coating performance was analyzed using the response surface methodology (RSM). The outcomes showed an inversely proportional relationship between the graphene concentration and hydrogen ingress. The permeation coefficient for pure PVDF was recorded as 16.74, which decreased to 14.23, 12.10, and 11.46 for 0.3, 0.5, and 1.0 wt% PVDF-G, respectively, with the maximum reduction of 31.6% observed at 1.0 wt%. ANOVA established statistical significance, along with indications of strong projection dependability. However, the inhibition reduction stabilized with increasing graphene concentrations, likely caused by nanoparticle agglomeration. The results support the notion of PVDF–graphene’s potential as a suitable coating for the transformation of pipelines for hydrogen transport infrastructure. This research will aid in the establishment of suitable contemporary barrier coating materials, which will enable the safe utilization of hydrogen energy in the current energy transportation grid.

## 1. Introduction

The need for energy security and sustainability has long fueled studies aimed at developing novel technologies to facilitate the production, transportation, and storage of energy in various forms. Traditionally, the focus was directed towards the enhancement of the oil and gas industry. However, the shift towards sustainable energy alternatives brought about new pathways to utilize previously developed technology and adapt it to the emerging energy sector [1]. Hence, to facilitate a shift towards sustainability, a transitional period is necessary, in which facilities and infrastructure based on newly adopted sources of energy will be required [2]. Hydrogen-based energy has become reputable due to its global abundance and highly beneficial byproducts—mainly water. While being advantageous, the light weight and versatile nature of hydrogen bring about a set of challenges in its transportation and storage. The present study focuses on an issue that is prevalent in the transportation of hydrogen. Pipelines are deemed the most favorable means due to their ability to transport large quantities carefully and affordably [3]. However, they bring a multitude of challenges, one of which is the embrittlement of steel pipelines due to hydrogen [4,5]. This is caused by hydrogen ingress into the lattice formation, which ultimately degrades the mechanical properties of the pipeline. Hydrogen embrittlement is commonly categorized into three main types: hydrogen environment embrittlement (HEE) [6], internal hydrogen embrittlement (IHE), and hydrogen reaction embrittlement [7]. HEE occurs upon exposure to a high-pressure gaseous hydrogen environment. IHE occurs due to hydrogen remnants in the metal, mainly introduced during manufacturing, processing, and welding. Nevertheless, HEE and IHE display similar effects, and both require external pressure for the embrittlement to take place. Both HEE and IHE share similar factors, which greatly impact the hydrogen environment, applied external pressure, and susceptibility. The hydrogen embrittlement behavior varies based on the environmental conditions and material characteristics; there are many contributing factors. Hydrogen pressure is directly proportional to the adsorption of atomic hydrogen per unit volume if the phase has not yet reached a saturated stage. As for the impact of temperature on hydrogen embrittlement, studies have shown that HEE typically occurs at room temperature, since, at lower temperatures, hydrogen atoms lack the energy to effectively diffuse into the material. Meanwhile, at higher temperatures, hydrogen is too mobile, and the trapping effect is overcome by the atoms [8]. Studies on the effects of the alloy composition on HEE have indicated that the nickel content is the main factor that dictates the effects of HE on austenitic steel; these studies collectively showed that nickel content above 12.5% resulted in negligible HE effects [9]. The principal mechanism is the degradation of materials, and there are a multitude of models that can reflect this mechanism individually or in combination. Hydrogen-enhanced decohesion (HEDE) suggests that degradation due to HE is a result of the decreasing cohesive strength of the atomic bonds in the material due to the presence of 1s hydrogen electrons in a 3d cell, causing a reduction in cohesive surface strength and reducing the fracture strength of the material [10]. The hydrogen-enhanced local plasticity (HELP) model attributes the degradation of a material due to hydrogen to the decrease in dislocation motion resistance, such as dislocation, which then acts as a carrier of plastic deformation across the lattice under lower stress [11]. Multiple approaches to mitigate hydrogen embrittlement have been studied; one popular approach is the application of coatings to reduce the permeability of hydrogen. Studies regarding the effectiveness of different types of coatings have been conducted. Further development of this field led to the utilization of nanoparticles in coating polymers, which proved to be effective in further reducing the hydrogen permeability—for example, by complicating the permeation path. Moreover, the addition of nanoparticles has improved the overall matrix strength [12]. In response to the above, this research aims to assess the potency of a PVDF–graphene composite coating as a permeation barrier. The study examines the performance of PVDF as a hydrogen-inhibiting coating, the function of graphene nanoparticles in improving coating performance, and the effect of the graphene concentration on hydrogen permeability. By addressing these aspects, this study aspires to contribute to the discovery of effective solutions to mitigate hydrogen embrittlement.

Several materials have been considered to mitigate hydrogen ingress in steel pipelines, each offering unique advantages and limitations. Metallic coatings such as tungsten display promising inhibition features but are restricted by the production complexity. Dielectric materials provide exceptional resistance owing to their compact oxide layers, yet their performance is typically limited to high temperatures. Polymer-based coatings have garnered attention due to their affordability, adaptability, and innately low hydrogen permeability. However, their predisposition to environmental degradation and limited mechanical robustness hamper their standalone use. Among polymers, fluoropolymers—particularly polyvinylidene fluoride (PVDF)—excel due to their hydrophobicity, stability, and robustness under variable conditions. Recent developments in nanomaterial incorporation propose that integrating graphene can further improve polymer-based coatings’ effectiveness by increasing the tortuosity and reducing the permeation paths. Hence, the focus of this study is PVDF–graphene coatings.

## 2. Sample Preparation and Testing Methods

To achieve an optimum PVDF–graphene nanocomposite coatings for metallic pipelines, the graphene concentration was set as the main variable, while the remaining parameters were kept constant. The selection of PVDF as the polymer matrix was based on this material’s chemical and thermal stability and ease of handling and processing. The graphene nanoparticles were sourced from Sigma-Aldrich, St. Louis, MO, USA, with the reported purity, lateral size, and thickness of >95%, 0.5–5 µm, and <3 layers, with electrical conductivity of 1000 s/m and a surface area greater than 500 m^2^/g. Graphene was selected for the purpose of enhancing the barrier capabilities.

### 2.1. Sample Selection and Preparation

In this study, 306 steel samples were selected as coating substrates. The substrates were cut into circular disks with a diameter of 5 cm using an electrical discharging machine (EDM) wire cutter, Mitsubishi Electric, Selangor, Malaysia. The surfaces of the samples were then polished using a three-stage grinding process, starting with 400-grit, followed by 600-grit and 1200-grit. The polished samples were immersed in an ultrasonic bath and then rinsed in acetone and air-dried to facilitate the removal of residues and debris. The PVDF polymer solution was based on a 10 wt% N-methyl-2-pyrrolidone (NMP)–PVDF ratio. The selection of NMP as the solvent was made to ensure optimal PVDF–graphene film formation, setting a benchmark for future efforts using green alternatives. Magnetic stirring for 30 min at 80 °C and 1500 rpm was used to achieve the homogenous mixing of the solution. The graphene concentrations were determined by means of the response surface methodology (RSM). The sample-specific concentrations of graphene were then added to the polymer solution, and it was mixed for an additional two hours under the same conditions. A set of 13 distinct formulations were produced: one sample of pure PVDF and twelve samples with variable graphene concentrations. Each solution was then stirred at 300 rpm and maintained at a temperature of 70 °C. This step was applied to preserve the solution’s properties until the coating commenced. The coating films were applied to the substrates using the spin coating method for 20 s, with a speed ranging from 500 to 2000 rpm; this cycle was repeated thrice for each sample. The samples were then cured in a vacuum oven at a temperature of 150 °C for 30 min.

### 2.2. Sample Characterization Tests

Upon the preparation of the coated samples, a series of characterization tests were performed. The purpose of these tests was to verify the functional applicability of the prepared coatings. Waters et al. [13] described the characterization of internal and external coatings used on steel pipelines and categorized the characterization tests designed for pipeline coatings into mechanical and corrosion tests. Some of the mechanical and corrosion tests and the relevant standards are shown in Table 1.

Abrasion resistance (ASTM D4060) testing is normally carried out using a Taber abrasion tester with a CS-10 wheel, Taber industries, Subang Jaya, Malaysia. with samples subjected to a 1000 g load for 500 cycles. The mass loss per cycle was determined using Equations (1) and (2):

(1) $$Mass\;Loss\;\left(mg\right)=W_{0}-W_{1}$$

(2) $$Abrasion\;Resistance=\frac{Mass\;loss\;(mg)}{Number\;of\;cycles}$$

Meanwhile, the pull-off adhesion test was conducted according to ASTM D454 using an Elcometer 108 pull-off adhesion tester, Elcometer, Puncak Alam, Malaysia, with 20 mm flat dollies. The epoxy adhesive was left to cure while exposed to uniform loads after the surface was cleaned to enable highly effective adhesion. In addition, the chemical degradation resistance test was carried out in accordance with NACE TM0174 to investigate the chemical durability of the samples. The samples were submerged for 30 days in a deionized water solution; the addition of 1 wt% HCl and 5 wt% NaCl served the purpose of emulating the saline and corrosive ocean environment. Post-test assessment involved mass loss and visual examination for blistering, discoloration, or delamination.

The coatings demonstrated enhanced abrasion resistance, with a mean weight loss of 10 mg per cycle (0.3% of total mass), exceeding the 0.38% loss reported in [17] for typical polymeric coatings. The pull-off adhesion results averaged 2.53 MPa, marginally lower than the 2.76 MPa achieved in [18], possibly due to the shorter curing period. In terms of chemical degradation, some samples exhibited exceptional resistance, with minimal discoloration or softening, while others experienced surface defects—these differences were attributed to filler dispersion inconsistencies.

## 3. Testing and Analysis Methods

### 3.1. Hydrogen Permeation Testing

**Figure 1:**
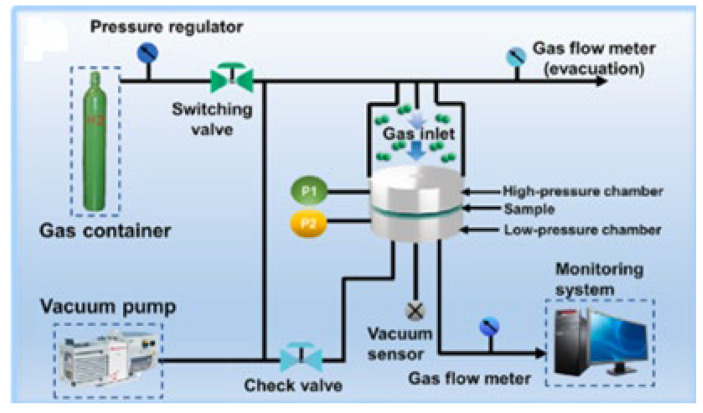
Schematic of membrane permeation cell system [19].

Considering the thermal limitations of polymer coatings, the high-temperature gaseous permeation test was discarded, leaving the membrane permeation cell as the most suitable means of evaluation. The coated samples were placed in an airtight chamber, and a pressure-regulated hydrogen tank supplied hydrogen to the inlet at a constant pressure of 35 bar, while the outlet was left at atmospheric pressure. Flow rates were then recorded every 10 min for a duration of 1 h for each sample. Figure 1 presents a schematic diagram of the membrane permeation chamber.

### 3.2. Response Surface Methodology

In design optimization, the response surface methodology (RSM) is a research approach in which a collection of statistical and mathematical techniques are used to model and analyze problems, where a response of interest is influenced by multiple input variables, with the goal of optimizing the response. Prior to conducting the permeation testing, the response surface methodology was used to obtain an execution plan for the coating process. The RSM i-optimal model was selected due to its flexibility in design, with the variable being the concentration of graphene, ranging from 0.1% to 1%, with a total of 12 runs. The designated graphene concentration range was chosen based on preceding studies, which suggested 1.0 wt% as the threshold beyond which performance stagnates or plummet due to dispersion challenges [20]. The RSM results are shown in Table 2.

### 3.3. Sample Characterization Analysis

**Figure 2:**
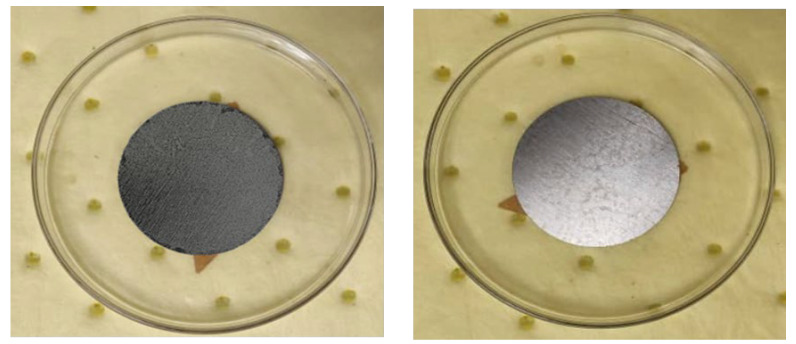
Samples demonstrating resistance to chemical degradation.

The abrasion test was conducted on three samples, with the mean weight loss per cycle determined to be 10 mg, amounting to 0.3% of the total sample weight. This was similar to the result reported by Garcia-Ruiz [17] for polymeric coatings of steel samples, which was 0.38%. The results provide strong insights into the enhanced performance of the nano-PVDF composite coating used in this study. In particular, this result strongly supports the pre-established notion that a polymer matrix’s mechanical properties are enhanced by virtue of nanoparticle inclusion. The ASTM D4151-based pull-off adhesion test yielded moderate results, with indications of cohesive failure at 2.53 Mpa [21]. In comparison, Hu et al. [18] achieved a slightly higher value of 2.761 MPa when testing ungalvanized PVDF-coated samples. In line with the aforementioned study, we allocated 120 h of curing time for the samples, allowing for enhanced surface adhesion, as well as addressing the potential for coating inhomogeneity, which in turn can create vulnerabilities on the coating surface. Figure 2 shows the samples prior to the chemical degradation test. The results showed contrasts in these samples, as one sample displayed discoloration and softening, while the other did not display such shortcomings, maintaining its properties with little to no pigmentation and strong mechanical features. Hong et al. [22] attributed such disparities and displays of failure in such tests to inconsistencies in filler dispersion.

## 4. Results and Analysis

### 4.1. Hydrogen Permeation

The permeation reduction performance of the samples was evaluated based on the gas transmission rate (*GTR*), which is used as a measure of the amount of gas passing through a screen over a specified time frame and under specified conditions. The *GTR* figures were then translated into permeability coefficient terms (*P*). The total gas permeated for each sample (*Q*) was measured at one hour (*t*), at the sample surface area (*A*), where areas of all samples amounted to 19.63 cm^2^. This information was used in Equation (3) to retrieve the *GTR* values. The results are shown in Table 3.

(3) $$GTR=\frac{Q}{A\cdot t}$$

Equation 4 was used to translate the GTR results into the permeation coefficients for each sample. The permeation coefficient (P) is measured in terms of the barrier and demonstrates that the permeation depends on the the sample thickness (L), allowing for a standardized and meaningful comparison among different materials. The sample thickness was held constant at 2 mm. Table 4 shows the computed permeation coefficient values for each concentration.

(4) $$P=GTR\;\cdot \;L\;\cdot \;10^{10}$$

### 4.2. Result Summary and Model Analysis

The collected and computed results were then fed back to the RSM statistical model, with the aim of evaluating the results in terms of the significance of the concentration for the permeation coefficient and the proposed model fit. As shown in Table 5, based on the results obtained, the quartic i-optimal model was the best-fitting model for the given data, with the highest combination of predicted and adjusted coefficients of determination, yielding an adjusted R^2^ value of 0.987 and a predicted R^2^ of 0.9661. The model also displayed higher statistical significance and low lack-of-fit values (Equation (5)).

(5) $$Y=11.79-1.62A+3.18A^{2}-0.13A^{3}-1.68A^{4}$$

In Table 6, the analysis of variance (ANOVA) results obtained from the RSM study are displayed. The results imply that the quartic model is a strong predictor regarding the impact of the graphene concentration on the hydrogen permeation coefficient. The model establishes a robust fit, with an F-value of 228.63 and a *p*-value of 2.81 × 10^−8^, verifying that the independent variable, i.e., the graphene concentration, bears significance regarding the response outcome. Hence, the correlation of the graphene concentration and permeation coefficient is non-linear. Nevertheless, the cubic term (A^3^) shows no significance regarding the response LOF of the F-value, with a value of 0.47, supporting that the model adequately reflects the experimental measurement fluctuations, attributing the bulk of the residual errors to pure errors and not model defects.

**Figure 3:**
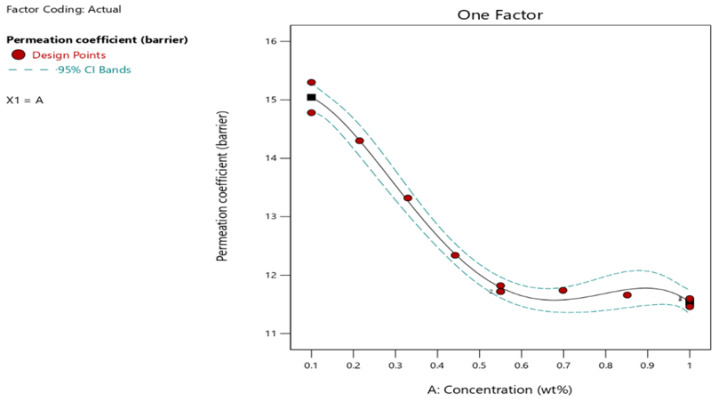
Permeation coefficient vs. graphene concentration: one-factor plot.

A strong correlation among the predicted and actual values of the permeation coefficient is demonstrated in the predicted vs. actual plot (Figure 3). The positioning of points adjacent to the diagonal line suggests that the model displays great predictive accuracy. Meanwhile, the one-factor response plot shows a clear declining trend in hydrogen permeation as the graphene concentration rises, strengthening its effectiveness as a barrier. Overall, both sets of results confirm the model’s strength and emphasize the potential of graphene as a practical hydrogen barrier.

**Figure 4:**
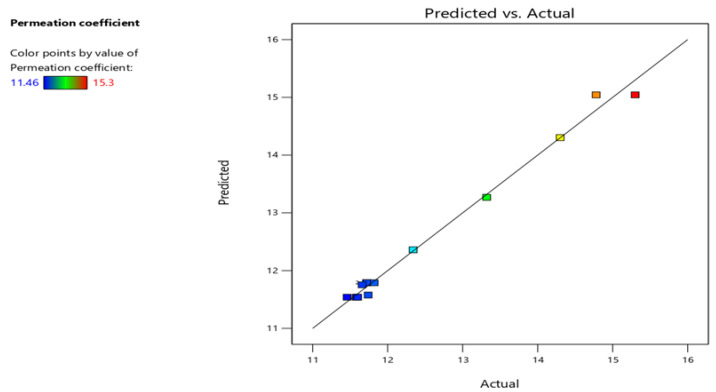
Permeation coefficient: predicted vs. actual result.

To ensure the reliability of this model, multiple residual diagnostic plots were created and analyzed. The normal probability distribution plot of residuals is illustrated in Figure 4. In ideal conditions, the data points should fall along the plot’s straight diagonal line. In the case of this model, the majority of the points adhere to the expected trend, except for a few mild outliers.

### 4.3. Effects of Graphene Concentration on Permeation

The adoption of PVDF contributed largely to the increase in the barrier’s inhibition performance. Despite this, its characteristic free volume acts as an uninterrupted medium for gas permeation upon adsorption; thus, the integration of graphene in the solution acts as a mitigator of gas flow in its free volume. The research by Yuan et al. [23] led to outcomes of a similar nature, where the prepared PEI-GO coatings exhibited heightened hydrogen barrier properties in PET substrate films. The induced effect of GO was attributed to the reduction of the free volume and the enhanced adhesion of the composite coating.

The findings of this study were further visualized in a bar chart, showing the permeation coefficient values for each sample, with pre-determined concentrations. These values can be compared to the permeation coefficient value of pure PVDF, which was 16.74 in this study. Two substantial permeation coefficient decrements were noted at 0.5% and 1%, which yielded barrier values of 11.72 and 11.46, respectively. Figure 3 shows the attained permeation coefficient reduction due to graphene incorporation, which visually reinforces the significance of the permeation coefficient.

It is evident that the observed results are strongly aligned with Fick’s first law of diffusion, which asserts that permeated flux across a membrane is directly proportional to the gradient concentration across it. As concluded previously, the integration of graphene nanoparticles into the PVDF matrix reduced the permeation allowance via an increase in the tortuosity of the permeation path, thus disrupting the progression of diffusion. This is, in principle, the driving factor for the inversely proportional relationship between the graphene concentration and the permeation coefficient.

Regarding time-dependent diffusion, Fick’s second law provides more insight into the non-linear relationship between the concentration and permeation. Since the second law includes position-dependent factors in the description of the change in concentration over time due to diffusion, the position factor is dependent on the thickness of the coating, which grows incrementally with the added graphene concentration. Given the non-linearity in the results, at the onset of the concentration increment, a significant reduction is observed. While this study did not directly assess the differences in film porosity among different graphene loading concentrations, primarily due to the performance-based approach adopted, the outcomes clearly imply an increase in film compactness and a decrease in film porosity by virtue of higher graphene concentrations in the coating.

However, past a specific threshold concentration value, the effect gradually plateaus, and further addition may lead to negative results. Such a response was encountered in [23,24,25], which was attributed to the agglomeration and possible aggregation of graphene beyond a threshold concentration, effectively reducing the diffusion path’s tortuosity. The dispersion and orientation of graphene in the polymer matrix provide an alternative explanation for the non-linearity observed, as it has been reported that the degree of mixing optimization strongly influences the permeation reduction. The previously mentioned studies [23,26] emphasize the importance and impacts of the nanocomposite matrix’s homogeneity in terms of increased barrier properties and note that spin coating application results in favorable graphene orientation. As noted in [27], graphene clustering in polymer matrices transpires due to strong van der Waals forces among nanosheets, leading to localized agglomeration and preventing uniform dispersion. These agglomerates reduce the tortuosity of the diffusion paths, diminishing the barrier’s effectiveness and leading to mechanical irregularities that lower adhesion and allow crack formation—eventually degrading the coating performance.

Upon comparative evaluation, the outcomes obtained are consistent with the reported literature regarding the performance of other graphene-based polymer composite coatings in hydrogen permeation inhibition. In agreement with our detected ≈31% decrease in hydrogen permeability (from 16.74 to 11.46 at 1.0 wt% graphene), comparable enhancements have been described in other polymer–graphene gas barriers. For instance, ref. [28] reported on graphene–PVA nanocomposite films, achieving a maximum penetrability reduction of 43% at 0.5 wt% graphene. Reported sources on graphene–SiC hybrid fluoropolymer coatings show a 75.66% reduction in hydrogen permeability [23]. Likewise, a layer-by-layer electrostatically assembled SPVDF-GO coating studied by Yuan et al. [23] significantly reduced the GTR values of hydrogen. This collectively enables a robust understanding of the performance of such coatings.

## 5. Conclusions

To assess the prospects of PVDF–graphene coatings, this study focused on the synthesis, evaluation, and analysis of the hydrogen permeation inhibition of such coatings. The outcomes indicate that the intrinsic free volume of pure PVDF coatings can be compensated for by the addition of a graphene nanofiller. These functional enhancements are attributed to the consequent increase in the tortuosity of the gas diffusion path, leading to a reduction in permeation capabilities. The findings establish an inverse relationship between the graphene concentration and hydrogen permeation, which is clearly evidenced in the decrease in the permeation coefficient from 16.74 to 11.46 for pure PVDF and 1.0 wt% graphene content. This lays a solid foundation for further studies on the long-term performance of such coatings, adding to the collective body of knowledge pertaining to the application of PVDF–graphene coatings, as well as coatings of a similar nature.

## Figures and Tables

**Table 1:** Sample characterization tests and applicable standards [13].

Test	Relevant Standard
Abrasion resistance	(ASTM D4060) [14]
Pull-off adhesion	(ASTM D4541) [15]
Resistance to chemical degradation	(NACE TM0174) [16]

**Table 2:** RSM experimental design.

Run	1	2	3	4	5	6	7	8	9	10	11	12	13
Graphene, wt%	0	0.214	0.55	0.422	0.1	0.698	0.1	0.329	0.55	1	1	0.851	1

**Table 3:** Total gas permeated and gas transmission rate results.

Run	Graphene Concentration wt%	Total Gas Permeated, Q (cm^3^/hour)	GTR (cm^3^/(cm^2^·s cmHg))
1	0	0.399	8.73 × 10^−9^
2	0.214663	0.341	7.15 × 10^−9^
3	0.55	0.282	5.91 × 10^−9^
4	0.442	0.294	6.17 × 10^−9^
5	0.1	0.364	7.65 × 10^−9^
6	0.6985	0.280	5.87 × 10^−9^
7	0.1	0.352	7.39 × 10^−9^
8	0.3295	0.317	6.66 × 10^−9^
9	0.55	0.279	5.86 × 10^−9^
10	1	0.279	5.79 × 10^−9^
11	1	0.281	5.90 × 10^−9^
12	0.8515	0.278	5.83 × 10^−9^
13	1	0.284	5.73 × 10^−9^

**Table 4:** Computed permeation coefficient results.

Run	Graphene Concentration wt%	Permeation Coefficient (Barrier)
1	0	16.74
2	0.214663	14.3
3	0.55	11.82
4	0.442	12.34
5	0.1	15.3
6	0.6985	11.74
7	0.1	14.78
8	0.3295	13.32
9	0.55	11.72
10	1	11.58
11	1	11.6
12	0.8515	11.66
13	1	11.46

**Table 5:** RSM fit results.

Model Order	Sequential *p*-Value	Lack-of-Fit *p*-Value	Adjusted R^2^	Predicted R^2^	Recommendation
Linear	9.9 × 10^−5^	0.00103	0.7399	0.6627	-
Quadratic	1.9 × 10^−6^	0.1896	0.9731	0.9588	-
Cubic	0.08252	0.1393	0.9702	0.9454	-
Quartic	0.0075	0.7188	0.9870	0.9661	Suggested
Fifth	0.5135	0.6362	0.9861	0.9445	-
Sixth	0.3217	0.9022	0.9864	0.9666	-

**Table 6:** RSM and ANOVA results.

Source	Sum of Squares	df	Mean Square	F-Value	*p*-Value	Significance
Model	22.43	4	5.61	228.63	<0.0001	Significant
A-concentration	1.3	1	1.3	53.18	<0.0001	-
A^2^	0.9808	1	0.9808	40	0.0002	-
A^3^	0.0065	1	0.0065	0.2656	0.6202	-
A^4^	0.3087	1	0.3087	12.59	0.0075	-
Residual	0.1962	8	0.0245			-
Lack of Fit	0.0428	3	0.0143	0.4657	0.7188	Not significant
Pure Error	0.1533	5	0.0307			
Corr. Total	22.62	12				

## Data Availability

The datasets used and/or analyzed during the current study are available from the corresponding author upon reasonable request.

## Abbreviations

The following abbreviations are used in this manuscript:

HE	Hydrogen Embrittlement
IHE	Internal Hydrogen Embrittlement
HEE	Hydrogen Environment Embrittlement
HELP	Hydrogen-Enhanced Local Plasticity
HEDE	Hydrogen-Enhanced Decohesion
PVDF	Polyvinylidene Fluoride
PVA	Polyvinyl Alcohol
RSM	Response Surface Methodology
GTR	Gas Transmission Rate
P	Permeability Coefficient
ANOVA	Analysis of Variance
NMP	N-Methyl-2-Pyrrolidone
NACE	National Association of Corrosion Engineers
